# Receptor protein tyrosine phosphatase delta is not essential for synapse maintenance or transmission at hippocampal synapses

**DOI:** 10.1186/s13041-020-00629-x

**Published:** 2020-06-17

**Authors:** Kyung Ah Han, Hee-Yoon Lee, Dongseok Lim, Jungsu Shin, Taek Han Yoon, Xinran Liu, Ji Won Um, Se-Young Choi, Jaewon Ko

**Affiliations:** 1 Department of Brain and Cognitive Sciences, Daegu Gyeongbuk Institute of Science and Technology (DGIST), 333 Techno Jungangdae-Ro, Hyeonpoong-Eup, Dalseong-Gun, Daegu, 42988 South Korea; 2 Core Protein Resources Center, DGIST, 333 Techno Jungangdae-Ro, Hyeonpoong-Eup, Dalseong-Gun, Daegu, 42988 South Korea; 3 Department of Physiology and Neuroscience, Dental Research Institute, Seoul National University School of Dentistry, Seoul, 03080 South Korea; 4 Department of Cell Biology, Yale University School of Medicine, New Haven, CT 06510 USA

**Keywords:** PTPδ, PTPσ, LAR-RPTP, Synapse maintenance, Synaptic transmission, Synaptic adhesion

## Abstract

Members of the leukocyte common antigen-related receptor protein tyrosine phosphatase (LAR-RPTP) family, comprising PTPσ, PTPδ and LAR, are key hubs for presynaptic assembly and differentiation in vertebrate neurons. However, roles of individual LAR-RPTP members have not been investigated using member-specific conditional knockout mice. Here, we show that loss of PTPδ had no overt effect on synapse development in mouse cultured hippocampal neurons. Moreover, loss of PTPδ in presynaptic CA1 hippocampal neurons did not influence neurotransmitter release in subicular pyramidal neurons, suggesting that PTPδ is not critical for presynaptic function in vivo. Our results demonstrate that PTPδ is not essential for synapse maintenance or transmission, at least in the mouse hippocampus, and underscore the importance of using sophisticated genetic approaches to confirm the roles of synaptic proteins.

## Introduction

Leukocyte common antigen-related receptor tyrosine phosphatases (LAR-RPTPs) are evolutionarily conserved synaptic organizers in vertebrate neurons [1–3]. LAR-RPTPs bind various postsynaptic ligands to organize multifarious synaptic adhesion pathways that are required for presynaptic assembly and a subset of postsynaptic functions [4–9]. Accumulating evidence from loss-of-function approaches, including constitutive knockout (KO) mice and short-hairpin RNA (shRNA)-mediated knockdown (KD), has lent support to this idea [2, 3, 10, 11]. PTPδ is strongly expressed in brains and constitutive PTPδ KO has been reported to impair learning, alter long-term synaptic plasticity, and cause abnormal dendritic arborization [12, 13]. Moreover, previous studies have reported that PTPδ KD produces pleiotropic effects on both presynaptic and postsynaptic processes [4, 6, 8, 14]. Although all three members of the vertebrate LAR-RPTP family (PTPσ, PTPδ and LAR) share similar structural, biochemical and ligand-binding profiles [3], individual LAR-RPTP members exhibit unique features. For example, PTPσ, but not PTPδ or LAR, binds to TrkC receptor tyrosine kinase, whereas PTPδ specifically binds to IL1RAPL1 (interleukin 1 receptor accessory protein like 1). Moreover, KD analyses in cultured hippocampal neurons have revealed dichotomous roles of PTPσ and PTPδ in regulating maintenance of excitatory and inhibitory synapse development, respectively [2, 5, 15, 16]. Although these findings clearly indicate the significance of LAR-RPTPs as important synaptic organizers, a more sophisticated approach using conditional KO (cKO) mice deficient in individual LAR-RPTP members is required to precisely evaluate the synaptic role of vertebrate LAR-RPTPs in vivo. A recent paper showed that all three LAR-RPTPs regulate postsynaptic responses mediated by N-methyl-D-aspartate receptors (NMDA-type glutamate receptors) through *trans*-synaptic mechanism(s), but this study did not examine the presynaptic role of individual LAR-RPTPs [17].

In the present study, we generated mutant mice carrying cKO alleles of PTPδ, and used a variety of experimental approaches employing both cultured hippocampal neurons and acute hippocampal slice preparations. We found that, in contrast to KD effects [4], conditional genetic deletion of PTPδ did not affect synapse numbers, synaptic transmission, or vesicular organization in presynaptic boutons. Moreover, loss of PTPδ in hippocampal CA1 pyramidal neurons did not affect neurotransmitter release at either excitatory or inhibitory synapses formed on subicular pyramidal neurons. Viewed together, our results suggest that PTPδ is not essential for synapse maintenance, synaptic transmission, or neurotransmitter release at hippocampal synapses.

## Materials and methods

### Cell culture

HEK293T cells were cultured in Dulbecco’s Modified Eagle’s Medium (DMEM; WELGENE) supplemented with 10% fetal bovine serum (FBS; Tissue Culture Biologicals) and 1% penicillin-streptomycin (Thermo Fisher) at 37 °C in a humidified 5% CO_2_ atmosphere. Cultured primary hippocampal neurons were prepared from embryonic day 17 PTPδ floxed mouse embryos.

### Animals

**Fig. 1:**
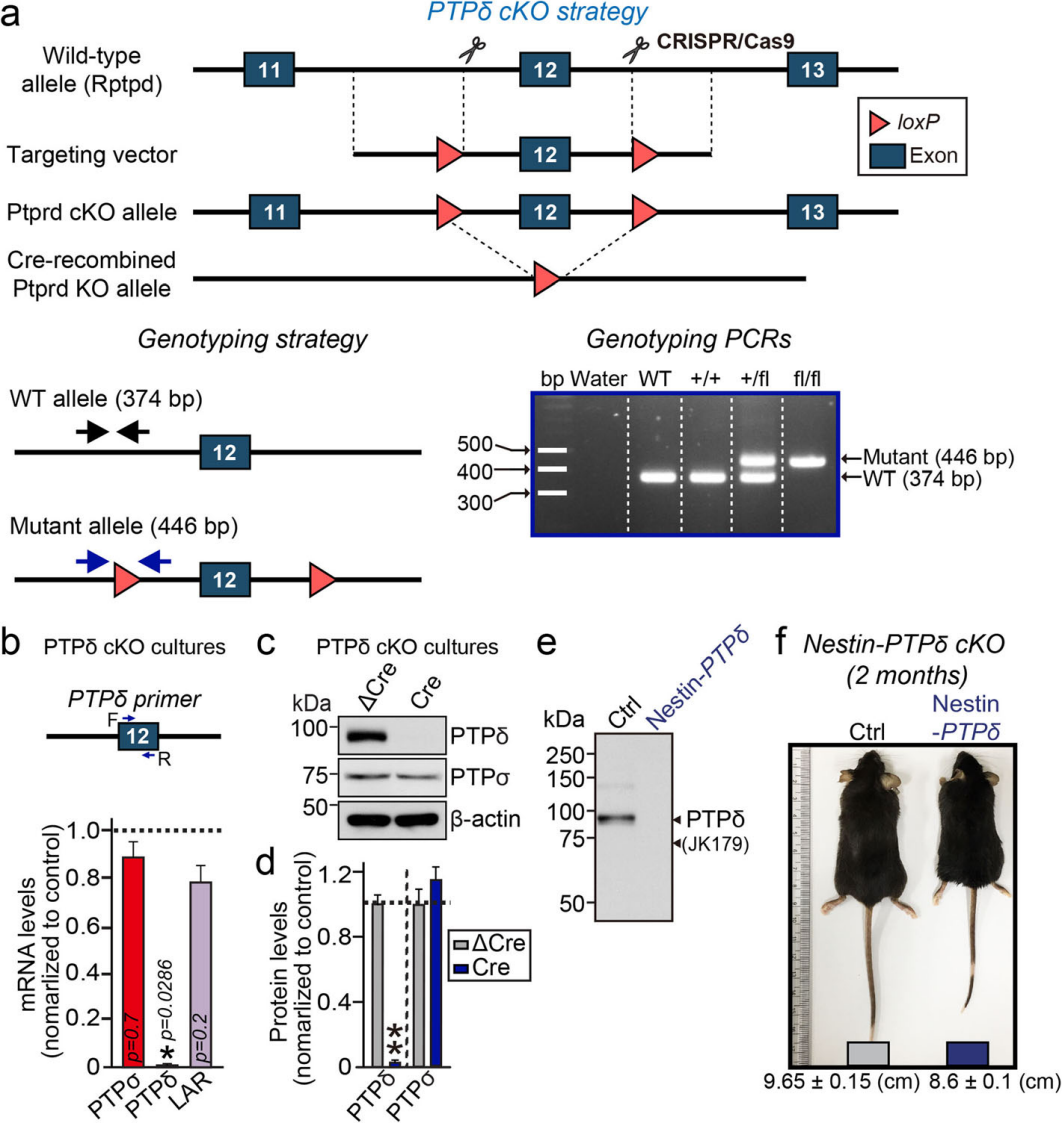
Generation of PTPδ-cKO mice. **a** cKO strategy for the PTPδ mouse line. *Upper:* Targeting of exon 12 of the PTPδ gene. *Bottom left:* Primer locations for WT and floxed alleles (arrows). Bottom r*ight:* PCR genotyping of WT and PTPδ^fl/fl^ mice. **b**, Quantitative RT-PCR analysis of neuronal mRNA. Relative levels of PTPσ, PTPδ, and LAR mRNAs were measured in cultured cortical neurons infected with lentiviruses expressing Cre-recombinase. Data are means ± SEMs (*n* = 3–4 independent experiments; *p < 0.05; Mann Whitney *U*-test). **c**, **d**, Representative immunoblot image (**c**) and quantitative analyses (**d**) of the level of PTPδ protein in cultured cortical neurons infected with lentiviruses expressing Cre-recombinase. Data are means ± SEMs (*n* = 6 independent experiments; **p < 0.01; Mann Whitney *U*-test). **e**, Representative immunoblot analysis of the level of PTPδ protein in brain homogenates from 8-week old control and PTPδ-cKO mice. Levels of PTPδ protein were measured in PTPδ^fl/fl^ mice (Ctrl) or PTPδ^fl/fl^ mice crossed with Nestin-Cre mice (Nestin-PTPδ). Arrows indicate band(s) that show immunoreactivity to PTPδ-specific (JK179) antibodies. **f**, An image illustrating the body size of littermate control (Ctrl) and Nestin-PTPδ mice at 2 months of age. Nestin-PTPδ mice were significantly smaller than age- and sex-matched Ctrl mice

The use and care of animals complied with the guidelines and protocols (DGIST-IACUC-17122104-01) for rodent experimentation approved by the Institutional Animal Care and Use Committee of DGIST under standard, temperature-controlled laboratory conditions. PTPδ conditional knockout mice were generated at Biocytogen Co., Ltd. (Beijing, China). Nestin-Cre (003771, Jackson Research Laboratories) mice were the gift of Dr. Albert Chen (DUKE-NUS, Singapore). Mice were kept on a 12:12 light/dark cycle (lights on at 9:00 am), and received water and food ad libitum*.* Floxed PTPδ (PTPδ^f/f^) was generated by flanking exon 12 with loxP sites (See Fig. 1a for details). Nestin-Cre driver line was crossed with *PTPδ*^f/f^ mice to generate pan-neuronal PTP*δ* knockout. Mice were maintained in the C57BL/6 N background. All experimental procedures were performed on male mice, using littermate control without Cre expression.

### Expression vectors

The following plasmids were kindly provided by other investigators: pAAV-hSyn-∆Cre-GFP and pAAV-hSyn-Cre-GFP from Dr. Thomas C. Südhof (Stanford University, Palo Alto, CA, USA; and FSW-∆Cre and FSW-Cre from Dr. Pascal S. Kaeser (Harvard University, Cambridge, MA, USA).

### Antibodies

Commercially obtained antibodies included: guinea pig polyclonal anti-VGLUT1 (Millipore; RRID: AB_2301751), rabbit polyclonal anti-VGLUT1 (Synaptic Systems; RRID: AB_887880), rabbit polyclonal anti-GABA_A_Rγ2 (Synaptic Systems; RRID: AB_2263066), mouse monoclonal anti-PSD-95 (clone K28/43; Neuromab; RRID: AB_2292909), mouse monoclonal anti-PTPσ (clone 17G7.2; MediMabs; RRID: AB_1808357), mouse monoclonal anti-CASK (clone K56A/50; NeuroMab; RRID: AB_2068730), rabbit polyclonal anti-Munc13-1 (Synaptic Systems; RRID: AB_887733), rabbit polyclonal anti-RIM-BP2 (Synaptic Systems; RRID: AB_2619739), mouse monoclonal anti-ELKS (Sigma-Aldrich; RRID: AB_2100013), mouse monoclonal anti-Synaptophysin (clone SVP-38; Sigma-Aldrich; RRID: AB_477523), mouse monoclonal anti-MAP2 (clone AP-20; Sigma-Aldrich; RRID: AB_477171), rabbit polyclonal anti-MAP2 (Abcam; RRID: AB_776174), mouse monoclonal anti-β-actin (clone C4; Santa Cruz Biotechnology; RRID: AB_626632), mouse monoclonal anti-GluN1 (clone 54.1; Millipore; RRID: AB_94946), rabbit polyclonal anti-Cav2.1 (Synaptic Systems; RRID: AB_2619841), and mouse monoclonal anti-Gephyrin (clone 3B11; Synaptic Systems; RRID: AB_887717). Rabbit polyclonal anti-liprin-α2 (RRID: AB_2810258) and rabbit polyclonal anti-liprin-α3 (RRID: AB_2810259) antibodies were gifts of Dr. Susanne Schoch-McGovern (Bonn, Germany); rat polyclonal anti-PTPδ antibody (RRID:AB_2810260) was the gift of Dr. Fumio Nakamura (Yokohama, Japan); rabbit polyclonal anti-pan-Shank (1172, RRID:AB_2810261), rabbit polyclonal anti-GluA1 (1193, RRID: AB_2722772), rabbit polyclonal anti-GluA2 (1195; RRID: AB_2722773), and rabbit polyclonal anti-Homer1 antibodies (1133, RRID, AB_2810985) were the gifts of Dr. Eunjoon Kim (KAIST, Korea).

### Neuron culture, immunostaining, imaging, and quantitation

Hippocampal and cortical mouse neuron cultures were prepared from embryonic day 17 (E17) mouse embryos, as described previously [18]. Mouse cultured neurons were seeded onto coverslips coated with poly-D-lysine (Sigma-Aldrich), and grown in Neurobasal medium supplemented with B-27 (Thermo Fisher), 0.5% FBS (WELGENE), 0.5 mM GlutaMAX (Thermo Fisher), and sodium pyruvate (Thermo Fisher). Cultured neurons were infected with lentiviruses at DIV3–4. For immunocytochemistry, cultured neurons were fixed with 4% paraformaldehyde/4% sucrose in PBS for 10 or 30 min at 4 °C, and permeabilized with 0.2% Triton X-100 in PBS for 10 or 30 min at 4 °C. Neurons were blocked with 3% horse serum/0.1% BSA in PBS for 15 min at room temperature and incubated with primary and secondary antibodies in blocking solution for 70 min at room temperature. The primary antibodies were used in these experiments included anti-VGLUT1 (Synaptic Systems; 1:700), anti-GABA_A_Rγ2 (Synaptic Systems; 1:500), anti-GluA1 (1193; 1:200), anti-Gephyrin (Synaptic Systems; 1:100), and anti-pan-Shank (1172; 1:200). Images of randomly selected neurons were acquired using a confocal microscope (LSM800, Carl Zeiss) with a 63 × objective lens; all image settings were kept constant during image acquisition. Z-stack images obtained by confocal microscopy were converted to maximal projections, and puncta size and the density of the indicated presynaptic marker proteins were analyzed in a blinded manner using MetaMorph software (Molecular Devices Corp.).

### Production of lentiviruses

Lentiviruses were produced by transfecting HEK293T cells with three plasmids—lentivirus vectors, psPAX2, and pMD2.G—at a 2:2:1 ratio. After 72 h, lentiviruses were harvested by collecting the media as previously described [4, 19].

### Production of adeno-associated viruses

HEK293T cells were co-transfected with the indicated AAV vectors, pHelper and AAV1.0 (serotype 2/9) capsids vectors. After 72 h, the transfected HEK293T cells were collected, and resuspended in PBS, and lysed by subjecting them to four freeze-thaw cycles in an ethanol/dry ice bath (7 min each) and a 37 °C water bath (5 min). The lysates were centrifuged and the supernatants were mixed with 40% polyethylene glycol and 2.5 M NaCl and centrifuged at 2000×g for 30 min. The cell pellets were resuspended in HEPES buffer (20 mM HEPES, 115 mM NaCl, 1.2 mM CaCl_2_, 1.2 mM MgCl_2_, and 2.4 mM KH_2_PO_4_, pH 8.0) to which was added an equal volume of chloroform. The mixture was centrifuged at 400×g for 5 min and concentrated three times with a Centriprep centrifugal filter (Cat. 4310, Millipore) at 1220×g (20 min each) and an Amicon Ultra centrifugal filter (Cat. UFC500396, Millipore) at 16,000×g for 30 min. AAVs were titered by treating 1 μl of concentrated, filter-sterilized AAVs with 1 μl of DNase I (AMPD1; Sigma) for 30 min at 37 °C to eliminate any contaminating plasmid DNA. After treatment with 1 μl of stop solution (50 mM ethylenediaminetetraacetic acid) for 10 min at 65 °C, 10 μg of protease K (Cat. P2308; Sigma) was added and the sample was incubated for 1 h at 50 °C. Reactions were stopped by heat inactivation at 95 °C for 20 min. The final virus titer was quantified by qRT-PCR. Empty AAV vector was used to generate a standard curve for qRT-PCRs targeting *GFP* sequences.

### qRT-PCRs

Cultured mouse cortical neurons were infected with recombinant lentiviruses at DIV4 and harvested at DIV13 for qRT-PCR using SYBR green qPCR master mix (TaKaRa). Total RNA was extracted from mouse cortical neurons using TRIzol reagent (Invitrogen) according to the manufacturer’s protocol. Briefly, cells in each well of a 12-well plate of cultured neurons were harvested and incubated with 500 μl TRIzol reagent at room temperature for 5 min. After phenol-chloroform extraction, RNA in the upper aqueous phase was precipitated. cDNA was synthesized from 500 ng of RNA by reverse transcription using a ReverTra Ace-α kit (Toyobo). Quantitative PCR was performed on a CFX96 Touch Real-Time PCR system (BioRad) using 0.5 μl of cDNA. The ubiquitously expressed β-actin was used as an endogenous control. The sequences of the primer pairs used were: mouse *Ptprs*, 5′-ATCAGAGAGCCCAAGGATCA-3′ (forward) and 5′-GCCACACACTCGTACACGTT-3′ (reverse); mouse *Ptprd*, 5′-CTCCTTGATCCCCATCTCTG–3′ (forward) and 5′-CAGGGCAGCCACTAAACTTC-3′ (reverse); and mouse *Ptprf*, 5′-CCCGATGGCTGAGTACAACA-3′ (forward) and 5′-CATCCCGGGCGTCTGTGA-3′ (reverse).

### Electron microscopy

E17 embryonic hippocampi of PTPδ floxed mice were seeded onto 18 mm coverslips at densities of 40,000 cells/well. The neurons were infected with lentiviral vectors expressing ΔCre or Cre at DIV4. At DIV14, cultured neurons were fixed in 2% glutaraldehyde, 0.1 M Na-cacodylate buffer, pH 7.4, for 1 h at room temperature and overnight at 4 °C. The cells were post-fixed in 0.5% OsO_4_ (osmium tetroxide), 0.8% K ferricyanide at room temperature for 60 min. All specimens were stained *en bloc* with 2% aqueous uranyl acetate for 30 min, dehydrated in a graded ethanol series up to 100%, embedded in Embed 812 resin (Electron Microscopy Science, PA), and polymerized overnight in a 60 °C oven. Thin sections (50–60 nm) were cut with a Leica ultramicrotome and post-stained with uranyl acetate and lead citrate. Sample grids were examined using a FEI Tecnai BioTWIN transmission electron microscope running at accelerating voltage of 80 kV. Images were recorded with a Morada CCD camera and iTEM (Olympus) software. This protocol allowed the unambiguous staining of membranes of synaptic vesicles as well as of pre- and post-synaptic compartments, resulting in accurate measurements of the nanoscale organization of the synaptic vesicles within nerve endings. To analyze synapse ultrastructure, the lengths of active zone and PSD, tethered vesicles, the membrane proximal vesicles, and total vesicle numbers were quantified using MetaMorph software (Molecular Devices). The numbers of total vesicles and docked vesicles were counted manually, and the distances from the active zone and the PSD to the vesicle center were measured. Vesicles located below 200 nm were considered membrane-proximal vesicles.

### Stereotaxic surgery and virus injections

4–5-week-old mice were anesthetized by intraperitoneal injection of 2% 2,2,2-tribromoethanol (Sigma), dissolved in saline, and secured in a stereotaxic apparatus. Viral solutions were injected using a Nanoliter 2010 Injector (World Precision Instruments), including a NanoFil syringe and 33 gauge needle, at a flow rate of 50 nl/min (injected volume, 500 nl). The coordinates used for stereotaxic injections targeting the ventral hippocampal CA1 were, relative to the bregma, anteroposterior (AP) -3.1 mm; medial–lateral (ML), ± 3.2 mm; and dorsal–ventral (DV), − 2.5 mm.

### In vitro and ex vivo electrophysiology

#### Electrophysiology of primary cultured neurons

Hippocampal neurons obtained from PTPδ floxed mice were infected on DIV4 with lentiviruses encoding Cre-EGFP or ΔCre-EGFP, followed by analysis at DIV13-16. Pipettes were pulled from borosilicate glass (o.d. 1.5 mm, i.d. 0.86 mm; Sutter Instrument), using a Model P^− 97^ pipette puller (Sutter Instrument). The resistance of pipettes filled with internal solution varied between 3 and 6 MΩ. The internal solution contained 145 mM CsCl, 5 mM NaCl, 10 mM HEPES, 10 mM EGTA, 0.3 mM Na-GTP, 4 mM Mg-ATP with pH adjusted to 7.2–7.4 with CsOH, and an osmolarity of 290–295 mOsmol/L. The external solution consisted of 130 mM NaCl, 4 mM KCl, 2 mM CaCl_2_, 1 MgCl_2_, 10 mM HEPES, and 10 mM D-glucose with pH adjusted to 7.2–7.4 with NaOH, and an osmolarity of 300–305 mOsmol/L. Whole-cell configuration was generated at RT using MPC-200 manipulators (Sutter Instrument) and a Multiclamp 700B amplifier (Molecular Devices). mEPSCs and mIPSCs were recorded at a holding potential of − 70 mV. Receptor-mediated synaptic responses were pharmacologically isolated by applying drug combinations of 50 μM picrotoxin, 10 μM CNQX, 50 μM D-APV and/or 1 μM tetrodotoxin. Synaptic currents were analyzed offline using Clampfit 10.5 (Molecular Devices) software.

#### Acute slice electrophysiology

Transverse hippocampal formation (300 μm) was prepared from 10 to 12-week-old male mice, as described [20]. The mice were anesthetized with isoflurane and decapitated, and their brains were rapidly removed and placed in ice-cold, oxygenated (95% O_2_/5% CO_2_), low-Ca^2+^/high-Mg^2+^ dissection buffer containing 5 mM KCl, 1.23 mM NaH_2_PO_4_, 26 mM NaHCO_3_, 10 mM dextrose, 0.5 mM CaCl_2_, 10 mM MgCl_2_, and 212.7 mM sucrose. Slices were transferred to a holding chamber in an incubator containing oxygenated (95% O_2_/5% CO_2_) artificial cerebrospinal fluid (ACSF) containing 124 mM NaCl, 5 mM KCl, 1.23 mM NaH_2_PO_4_, 2.5 mM CaCl_2_, 1.5 mM MgCl_2_, 26 mM NaHCO_3_, and 10 mM dextrose at 28–30 °C for at least 1 h before recording. After > 1 h incubation in ACSF, slices were transferred to a recording chamber with continuous perfusion (2 ml/min) by ACSF oxygenated with 95% O_2_/5% CO_2_ at 23–25 °C. All recordings were performed on pyramidal neurons in the subiculum identified by their size and morphology. Virus-infected neurons were identified by GFP fluorescence. Patch pipettes (4–6 MΩ) were filled with a solution containing 135 mM K-Gluconate, 8 mM NaCl, 10 mM HEPES, 2 mM ATP-Na and 0.2 mM GTP-Na (for measuring EPSC-PPRs); 130 mM Cs-MeSO_4_, 0.5 mM EGTA, 5 mM TEA-Cl, 8 mM NaCl, 10 mM HEPES, 1 mM QX-314, 4 mM ATP-Mg, 0.4 mM GTP-Na and 10 mM phosphocreatine-Na_2_ (for measuring IPSC-PPRs). The extracellular recording solution consisted of ACSF and holding at 0 mV when measuring IPSC-PPRs and supplemented with picrotoxin (100 μM) for measuring EPSC-PPRs. Evoked synaptic responses were elicited by stimulation (0.2 ms current pulses) using a concentric bipolar electrode (for CA1-subiculum synapses) placed 200–300 mm in front of postsynaptic pyramidal neurons at intensities that produced 40–50% of the maximal E/IPSC amplitude. Recordings were obtained using a Multiclamp 700B amplifier (Molecular Devices) under visual control with differential interference contrast illumination on an upright microscope (BX51WI; Olympus). Data were acquired and analyzed using pClamp 10.7 (Molecular Devices). Signals were filtered at 3 kHz and digitized at 10 kHz with DigiData 1550 (Molecular Devices).

### Data analysis and statistics

All data are expressed as means ± SEM. All experiments were repeated using at least three independent cultures, and data were statistically evaluated using a Mann-Whitney *U* test, analysis of variance (ANOVA) followed by Tukey’s post hoc test, Kruskal-Wallis test (one-way ANOVA on ranks) followed by Dunn’s pairwise post hoc test, or paired two-tailed t-test, as appropriate. Prism7.0 (GraphPad Software) was used for analysis of data and preparation of bar graphs. *P-*values < 0.05 were considered statistically significant (individual *p*-values are presented in figure legends).

### Availability of data and materials

All data generated or analyzed during this study are included in this published article. Any additional information related to the current study is available from the corresponding author on reasonable request.

## Results

### Generation of PTPδ-cKO mice

To determine the in vivo synaptic functions of PTP*δ*, we generated transgenic mice with a deletion of *PTPδ* by crossing *PTPδ*^f/f^ mice (with exon 12 flanked by loxP sites) with a Cre recombinase driver line in which Cre is expressed under control of the Nestin promoter (Nestin-Cre) (Fig. 1a). To assess the effects of endogenous PTPδ deletion, we induced a global loss of PTPδ in all neurons by infecting cultured hippocampal neurons from PTPδ-cKO mice at 3–4 days in vitro (DIV) with lentiviruses expressing EGFP-fused nuclear Cre-recombinase, which exhibits high infection efficiency. Infection with a non-functional mutant version of Cre-recombinase (ΔCre) was used as a control. Global expression of Cre-recombinase caused a nearly complete and specific loss of PTPδ mRNA expression in PTPδ cKO neurons analyzed at DIV13–14, without affecting PTPσ or LAR mRNA (Fig. 1b). Immunoblotting also confirmed complete elimination of PTPδ protein expression in cultured cortical neurons (Fig. 1c, d) and brain lysates (Fig. 1e) of PTPδ cKO mice. PTPδ cKO mice, in which PTPδ was deleted from the entire brain, were viable and fertile, but showed a modest reduction in body size (Fig. 1f). There were no alterations in expression of various classes of synaptic proteins linked to presynaptic functions (Fig. S1).

### Loss of PTPδ does not reduce the number of excitatory or inhibitory synapses

**Fig. 2:**
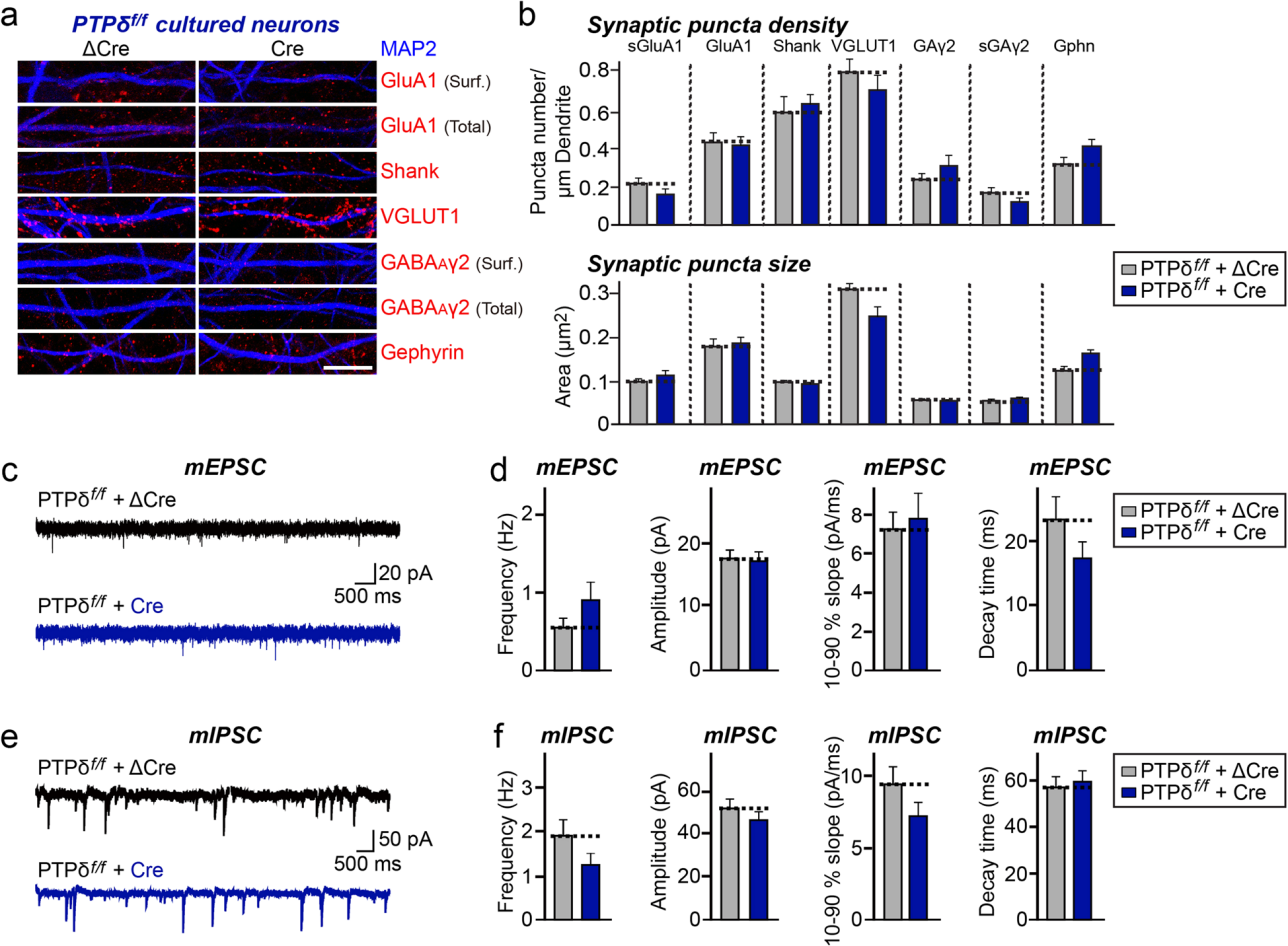
Loss of PTPδ does not affect synapse development or synaptic transmission in cultured hippocampal neurons. **a**, PTPδ cKO in cultured hippocampal neurons does not affect excitatory or inhibitory synapse density. Double-immunofluorescence analysis of MAP2 (blue) and the indicated synaptic markers (red) in mature cultured neurons (DIV14) derived from PTPδ^f/f^ mice, infected with lentiviruses expressing ΔCre or Cre at DIV3. Synaptic markers assayed include the excitatory synaptic markers, surface GluA1 (sGluA1), total GluA1, Shank and VGLUT1, and the inhibitory synaptic markers, surface GABA_A_γ2 (sGAγ2), total GABA_A_γ2, and gephyrin (Gphn). Scale bar: 10 μm. **b**, Quantification of images in (**a**), measuring the density of the indicated synaptic markers. Data are means ± SEMs (n denotes the number of analyzed neurons; ∆Cre/sGluA1, *n* = 14; Cre/sGluA1, n = 14; ∆Cre/GluA1, n = 14; Cre/GluA1, *n* = 15; ∆Cre/Shank, *n* = 21; Cre/Shank, n = 21; ∆Cre/VGLUT1, *n* = 16; Cre/VGLUT1, n = 16; ∆Cre/sGABA_A_Rγ2, n = 16; Cre/sGABA_A_Rγ2, *n* = 17; ∆Cre/GABA_A_Rγ2, *n* = 10; Cre/GABA_A_Rγ2, *n* = 9; ∆Cre/Gephyrin, n = 16; and Cre/Gephyrin, n = 16, n = 16; Mann Whitney *U*-test). **c**, **d**, Representative mEPSC traces (**c**) and quantification of frequencies, amplitudes and kinetics (**d**) of mEPSCs recorded from cultured hippocampal neurons derived from PTPδ^f/f^ mice infected with lentiviruses expressing ΔCre or Cre. Data are means ± SEMs (n denotes the number of analyzed neurons; ΔCre, *n* = 19; Cre, *n* = 13; unpaired *t*-test). **e**, **f**, Representative mIPSC traces (**e**) and quantification of frequencies, amplitudes and kinetics (**f**) of mIPSCs recorded from cultured hippocampal neurons derived from PTPδ^f/f^ mice infected with lentiviruses expressing ΔCre or Cre. Data are means ± SEMs (n denotes the number of analyzed neurons; ΔCre, *n* = 18; Cre, n = 16; unpaired *t*-test)

To evaluate the synaptic role of PTPδ, we infected cultured hippocampal PTPδ floxed neurons with lentiviruses expressing either ΔCre (control) or wild-type Cre recombinase at DIV3–4, and immunostained neurons for various excitatory and inhibitory synaptic markers at DIV14 (Fig. 2a, b). Surprisingly, this immunocytochemical analysis showed no significant change in the density or size of excitatory or inhibitory synaptic puncta (Fig. 2a, b). These results are in stark contrast to previous studies of the effect of PTPδ KD, which reported a specific reduction in inhibitory synapse numbers [4, 15].

### Loss of PTPδ does not impair synaptic transmission

To assess whether PTPδ loss affects synaptic transmission, we performed an electrophysiological assessment of cultured hippocampal neurons (Fig. 2c*–*f). Cultured hippocampal PTPδ floxed neurons were infected with lentiviruses expressing either ΔCre (control) or Cre recombinase at DIV3, and miniature excitatory postsynaptic currents (mEPSCs) and miniature inhibitory postsynaptic currents (mIPSCs) were recorded at DIV14–16 (Fig. 2c*–*f). In line with results from imaging experiments (Fig. 2a, b), lentivirus-mediated global loss of PTPδ had no effect on baseline synaptic transmission at excitatory or inhibitory synapses (Fig. 2c*–*f). Again, these findings are different from previous reported results for PTPδ KD showing specific reductions in inhibitory synaptic transmission [4].

### Loss of PTPδ does not alter synaptic vesicle tethering or active zone (AZ) architectures

**Fig. 3:**
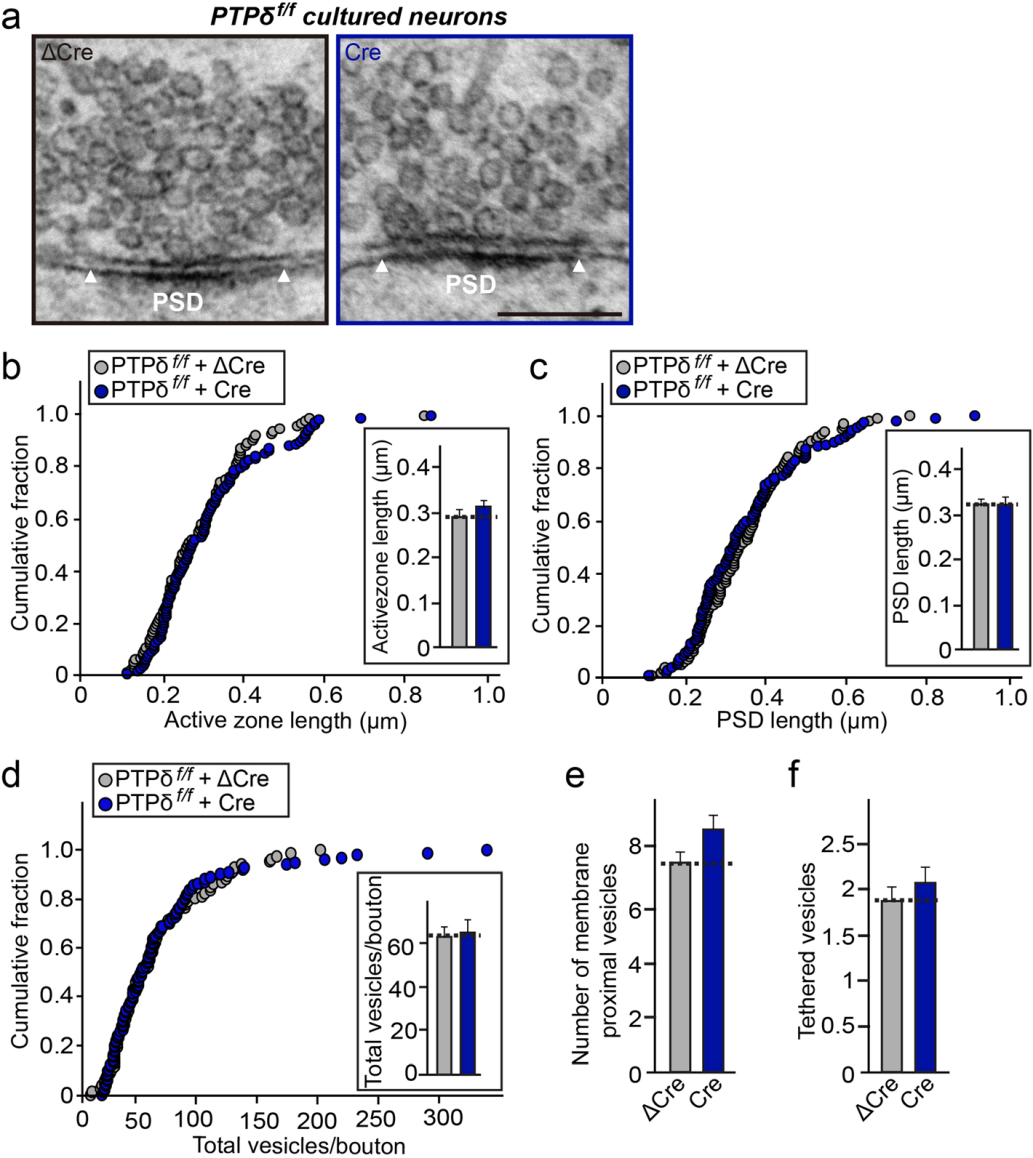
Loss of PTPδ does not induce abnormal organization of synapse structures in cultured hippocampal neurons. **a**, Representative electron micrographs of cultured hippocampal neurons from PTPδ^f/f^ mice infected with lentiviruses expressing ΔCre (control) or Cre. **b**, **c**, PTPδ deletion has no effect on the length of synaptic membranes. Cumulative distributions of the lengths of AZ (**b**) and PSD (**c**) for the indicated genotypes. Data are means ± SEMs (n denotes the number of analyzed neurons; ΔCre, *n* = 88; Cre, *n* = 105; Mann Whitney *U*-test). **d–f**, Total number of vesicles per bouton (**d**), membrane-proximal vesicles (**e**), and membrane-tethered vesicles (**f**) in control and PTPδ-deficient synapses. Data are means ± SEMs (n denotes the number of analyzed neurons; ΔCre, n = 88; Cre, n = 105; Mann Whitney *U*-test)

To further probe whether PTPδ loss affects synaptic structures, we imaged presynaptic terminals and postsynaptic densities of chemically fixed, cultured hippocampal neurons from PTPδ floxed mice infected with lentiviruses expressing either ΔCre (control) or Cre recombinase using transmission electron microscopy (TEM), as previously described [21] (Fig. 3). TEM analyses of cultured neurons showed that length of active zone (AZ) and postsynaptic density (PSD) were similar between PTPδ-deficient and control presynaptic terminals (Fig. 3a*–*c). Moreover, PTPδ-deficient and control presynaptic terminals contained similar numbers of total vesicles, and that the distribution of vesicles in PTPδ-deficient terminals was similar that in control presynaptic terminals (Fig. 3d). In addition, the numbers of membrane-proximal vesicles (defined as vesicles < 100 nm from the presynaptic AZ membrane) in PTPσ-deficient presynaptic terminals were also comparable to those in control presynaptic terminals (Fig. 3e). Moreover, the number of tethered vesicles, defined as vesicular structures docked at presynaptic AZ membranes, was also similar (Fig. 3f). Overall, these results suggest that PTPδ is not crucial for maintaining the structural organization of either presynaptic AZs or PSDs in hippocampal neurons.

### Loss of PTPδ in presynaptic neurons does not affect neurotransmitter release at postsynaptic target neurons

**Fig. 4:**
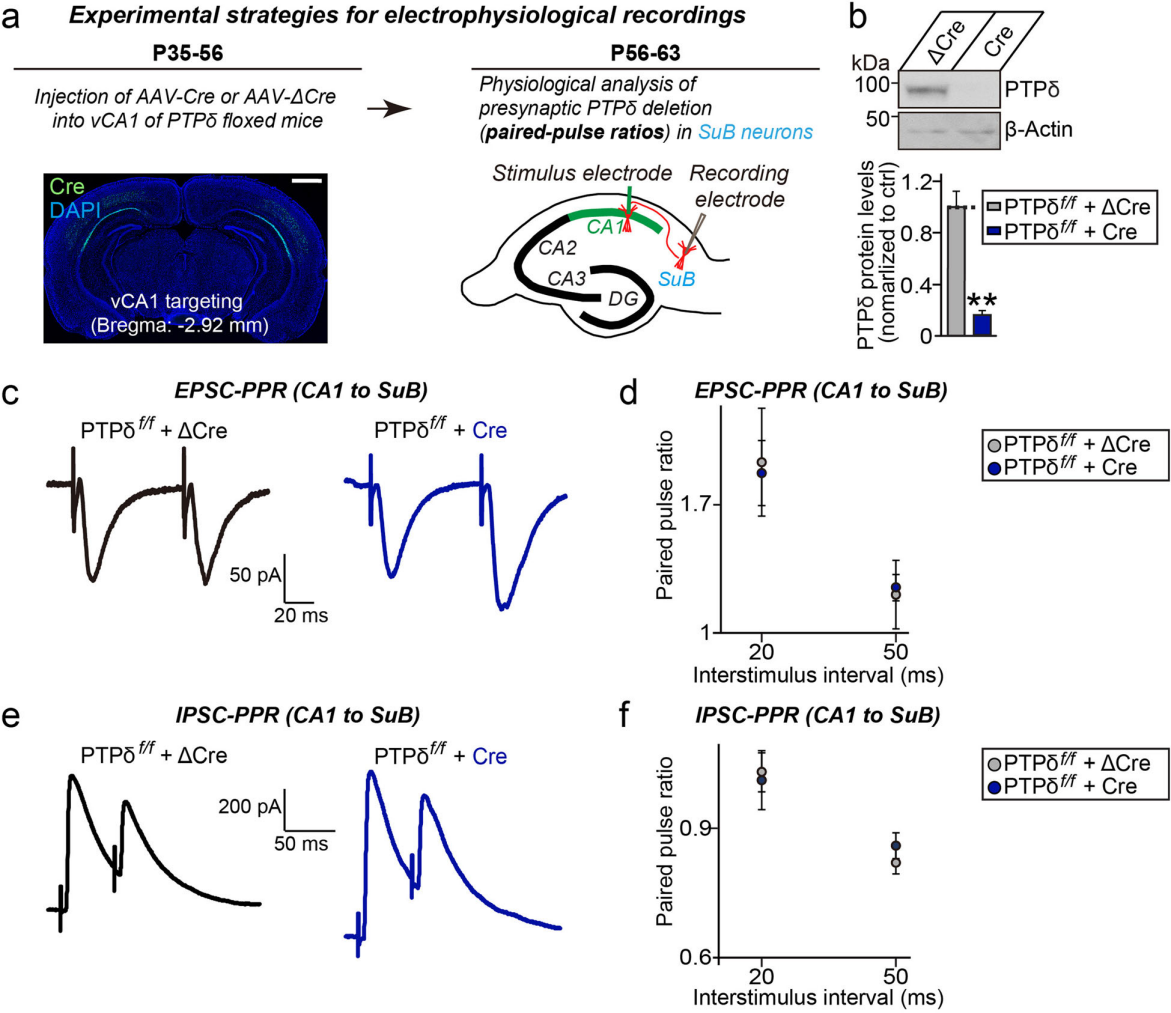
Presynaptic deletion of PTPδ in hippocampal CA1 neurons does not affect neurotransmitter release at excitatory or inhibitory synapses in postsynaptic subicular (SuB) pyramidal neurons. **a**, Experimental strategies for electrophysiological recordings in hippocampal SuB neurons innervated by PTPδ-deficient CA1 neurons. Representative coronal section showing EGFP expression after AAV-Cre injections into the ventral CA1 region of PTPδ floxed mice. Scale bar, 1 mm. **b**, Analysis of AAV-Cre expression in the hippocampal CA1 region of PTPδ-cKO mice. Representative immunoblot analyses (**top**) with PTPδ antibodies showing deletion of PTPδ protein in vivo. Infected mouse brain lysates were collected after stereotactic injection of AAV-Cre or AAV-ΔCre (Control) and immunoblotted with anti-PTPδ antibodies. Anti-β-actin antibodies were used as normalization controls. Quantitative analysis (**bottom**) of immunoblotting experiments. Data are means ± SEMs (*n* = 5 mice/group; **p < 0.01; Mann Whitney *U*-test). **c**, **e**, Representative traces of paired-pulse ratios (PPRs) of EPSCs (**c**) and IPSCs (**e**) in hippocampal CA1-SuB synapses at two different interstimulus intervals (20 and 50 ms). **d**, **f**, EPSC-PPRs (**d**) and IPSC-PPRs (**f**) in hippocampal CA1-SuB synapses as a function of the indicated interstimulus intervals. Data are means ± SEMs (n denotes the number of analyzed neurons; ΔCre [EPSC-PPR], *n* = 6; Cre [EPSC-PPR], *n* = 7; ΔCre [IPSC-PPR], *n* = 16; and Cre [IPSC-PPR], *n* = 12; two-tailed student’s *t*-test)

Lastly, we sought to address the role of PTPδ in regulating presynaptic neurotransmitter release in acute hippocampal slice preparation. In these experiments, we targeted hippocampal CA1-to-subicular synapses for analyses because of its amenability for presynaptic manipulations by stereotactic injection of viruses into the hippocampal CA1 region [22, 23]. We stereotactically injected adenoassociated viruses (AAVs) expressing either ΔCre (Control) or Cre recombinase into hippocampal CA1 region of *PTPδ*^f/f^ mice. PTPδ KO in vivo was validated by quantitative immunoblot analyses using hippocampal CA1 tissue lysates of AAV-injected mice (Fig. 4a, b). We then recorded the paired-pulse ratios (PPRs) of excitatory (EPSC-PPRs) or inhibitory (IPSC-PPRs) postsynaptic currents in subicular pyramidal neurons. Again, neither EPSC-PPRs nor IPSC-PPRs were altered in subicular neurons from Cre-infected *PTPδ*^f/f^ mice (Fig. 4c*–*f), suggesting that PTPδ is not essential for neurotransmitter release at excitatory or inhibitory synapses in the hippocampus in vivo.

## Discussion

There is extensive literature support for the idea that PTPδ is important for synapse development [2, 24]. The current study using PTPδ floxed mice infected with Cre recombinase, in conjunction with imaging, electrophysiology and electron microscopy, clearly demonstrated that conditional deletion of PTPδ does not significantly affect most synaptic parameters. These results were quite surprising and unexpected. Some discrepancies between the results of this study and previous studies—even including those by our laboratory—may be attributable to differences in developmental changes caused by constitutive KO and the well-documented off-target effects of RNA interference. Secondary effects triggered by KD may also have contributed to previously observed severe phenotypes; for example, PTPδ KD induced PTPσ upregulation in neurons, and vice versa, whereas PTPδ KO did not [4] (Fig. S1). It is also plausible that functional redundancies between LAR-RPTP proteins contribute to masking PTPδ loss-induced phenotypes in our experimental approaches. A recent study employing triple-cKO mice lacking all three LAR-RPTPs showed that, overall, the effects of LAR-RPTP triple cKO on cultured hippocampal neurons and hippocampal Schaffer-collateral synapses were marginal [17]. Remarkably, this latter study showed that triple LAR-RPTP cKO in presynaptic CA3 neurons strongly suppressed NMDA-type postsynaptic responses in postsynaptic CA1 neurons [17]. Our recent results using PTPσ-cKO mice did not reveal a similar *trans*-synaptic effect on postsynaptic responses [25]. Thus, testing whether presynaptic PTPδ is mainly involved in contributing to the reported *trans*-synaptic regulation of postsynaptic responses is warranted. However, it is equally likely that all three LAR-RPTPs are required for operation of this process in vivo. Regardless of precise answer(s), the current study indicates the importance of confirmatory analyses using a sophisticated system and approaches in elucidating functions of synaptic proteins in vivo.

As limitations, it could be argued that the approaches employed in the current study are not sufficiently sensitive to detect subtle changes in synapse properties. Moreover, the selection of experimental preparations may preclude detection of synaptic roles of PTPδ at in vivo synapses. Although PTPδ mRNA is widely distributed across rodent brain areas [26], it remains to be determined whether endogenous PTPδ protein is widely expressed or is restricted to a subset of certain neuron types or neural circuits. The lack of this information still hinders our ability to fully address the synaptic roles of PTPδ proteins. Development of effective PTPδ-specific antibodies suitable for high-resolution in vivo imaging is also warranted, as has similarly been shown for other cell-adhesion proteins [27]. With such tools and information in hand, it should be possible to use the PTPδ-cKO mice developed in the current study to unravel the canonical and non-canonical roles of PTPδ at appropriate in vivo neural circuits. Doing so may provide a better understanding of how LAR-RPTPs act as multivalent signaling platforms in presynaptic neurons.

## Supplementary information

**Additional file 1: Figure S1.** Quantitative immunoblot analyses of PTPδ-deficient mouse brains.a, Representative images of immunoblot analysis using brain lysates from Nestin-*PTPδ* mice (*n* = 4 mice/group). b, Quantitative immunoblot analysis of PTPs, AZ proteins, and PSD proteins from control and Nestin-*PTPδ* mice. Data are means ± SEMs (n = 4 mice/group; *p < 0.05; Mann Whitney *U*-test).

## Data Availability

The datasets used and/or analysed during the current study are available from the corresponding author on reasonable request.

## Abbreviations

AAV: Adeno-associated virus
cKO: Conditional knockout
EPSC: Excitatory postsynaptic current
IPSC: Inhibitory postsynaptic current
KD: Knockdown
LAR-RPTP: Leukocyte common antigen-related receptor protein tyrosine phosphatase
NMDA: N-*methyl*-D-aspartate
PPR: Paired-pulse ratio
TEM: Transmission electron microscopy
VGLUT1: Vesicular glutamate transporter 1
