# Supplementary figures and images for: Receptor protein tyrosine phosphatase delta is not essential for synapse maintenance or transmission at hippocampal synapses
Source: Mol Brain. 2020 Jun 17;13:94. doi: 10.1186/s13041-020-00629-x (PMC7301452; doi:10.1186/s13041-020-00629-x)

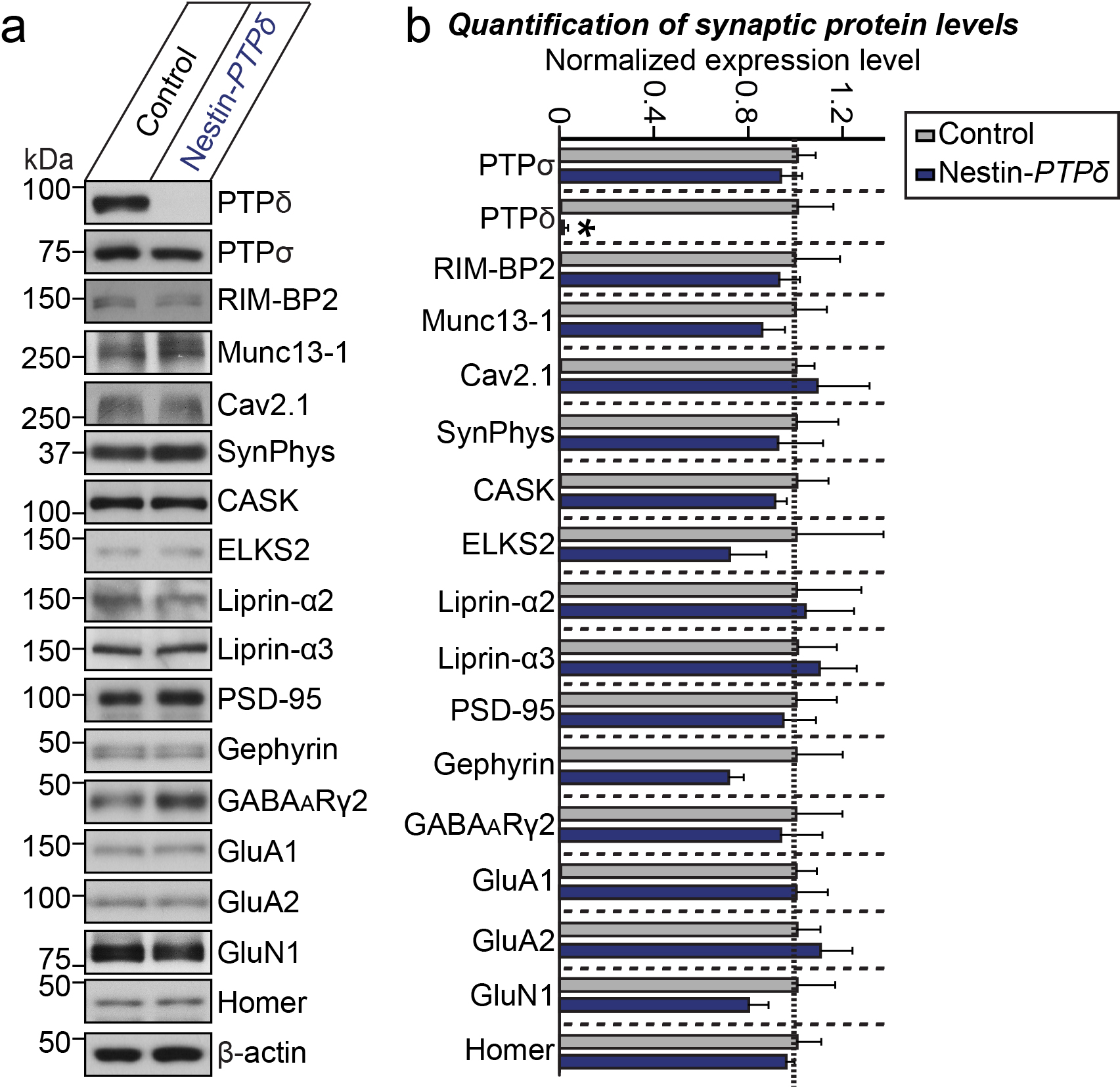

Supplement: Supplementary file 1 — Additional file 1: Figure S1. Quantitative immunoblot analyses of PTPδ-deficient mouse brains.a, Representative images of immunoblot analysis using brain lysates from Nestin-PTPδ mice (n = 4 mice/group). b, Quantitative immunoblot analysis of PTPs, AZ proteins, and PSD proteins from control and Nestin-PTPδ mice. Data are means ± SEMs (n = 4 mice/group; *p < 0.05; Mann Whitney U-test). [file 13041_2020_629_MOESM1_ESM.jpg]
